# The Perioperative Challenges of Major Lower Extremity Amputation and the Impact of Regional Anesthesia on Morbidity, Mortality, and Pain Management: A Narrative Review

**DOI:** 10.7759/cureus.78983

**Published:** 2025-02-14

**Authors:** Abdulaziz H. Algain

**Affiliations:** 1 Division of Anesthesia Services, King Abdulaziz University Hospital, Jeddah, SAU; 2 Department of Anesthesia and Critical Care, Faculty of Medicine, King Abdulaziz University, Jeddah, SAU

**Keywords:** chronic post-surgical pain, early recovery after surgery, lower extremity amputation, perineural local anesthetic infusion, peripheral nerve blocks, phantom limb pain, regional anesthesia, residual limb pain, transitional pain service

## Abstract

Limb amputation can impose severe burdens on the individual and society. Regardless of the underlying cause of amputation, pain management is challenging and may impact patients’ recovery and quality of life. Individuals undergoing major lower extremity amputation (MLEA) face significant perioperative risk. Therefore, anesthesiologists must meticulously customize their anesthetic approach. Regional anesthesia (RA) provides numerous physiological advantages over general anesthesia (GA) and is essential for pain management in orthopedic surgeries, standing as an excellent anesthesia method for high-risk patients and being fundamental in multimodal analgesia. This narrative review is an attempt to enhance understanding of different pain phenomena following limb amputation and to provide a critical synthesis of the existing evidence concerning the efficacy and impact of RA on morbidity, mortality, and pain management following MLEA, aiming to shed light on areas that have not received enough attention within these aspects and subsequently serve as a guide for future research. Despite the persistent controversy regarding the comparative mortality rates associated with RA versus alternative anesthetic methods for MLEA, several studies praise their efficacy in pain management and in mitigating adverse perioperative outcomes. Given that much of this data originates from retrospective studies, randomized multicenter prospective trials remain essential to validate their actual efficacy. A comprehensive analysis of the impact of RA on healthcare costs and resources related to MLEA is necessary to determine its correlation with cost reduction, decreased hospital stays, improved resource allocation, and increased patient satisfaction.

## Introduction and background

With around 185,000 major lower extremity amputation (MLEA) cases occurring in the United States each year, millions of people today live with major limb amputations, and projections indicate that by the year 2050, there will be about 3.6 million people living with limb loss [1,2]. The rise in amputation rates poses grounds for concern over the subsequent pain phenomena, perioperative adverse events, and their associated costs for healthcare. This review will examine current advancements in regional anesthesia (RA) designed to tackle these concerns. It will also highlight research gaps, primarily regarding the superiority of RA over other forms of anesthesia in reducing morbidity and mortality. 

The difficult nature of post-amputation pain management is owed to the severe comorbidities and the significant extent of tissue damage. Additionally, the locations of centers responsible for generating pain are varied and include peripheral, spinal, and cortical regions. Moreover, the nociceptive and neuropathic pain components lead to a complex and highly diverse array of postoperative pain syndromes [3]. Poor acute postsurgical pain management can induce chronic pain. One year following amputation or thoracic surgery, chronic postsurgical pain rates can reach 50-70 % [4].

RA offers various physiological benefits compared to general anesthesia (GA), including attenuating the stress response, enhancing the blood flow to the stump, reducing systemic effects, and delivering similar or better postoperative analgesia while sparing opioids [5,6]. Although numerous studies indicate that RA techniques for lower extremity amputations yield superior pain management, fewer pulmonary complications, decreased overall perioperative adverse events, shorter hospital stays, and lower rates of intensive care unit (ICU) admissions and reoperations, most studies failed to establish a correlation between reduced mortality rates and the application of RA compared to other anesthesia methods. As a secondary observation, there is a lack of research examining the economic and resource implications of RA specifically for lower extremity amputations. Continued investigation into this significant issue deserves attention, particularly given the increasing incidence of MLEA.

This narrative review addresses considerations surrounding MLEA and the resultant pain phenomena, as well as other major perioperative adverse events. It briefly describes the anatomy of the nerve network relevant to MLEA, outlines the most up-to-date, evidence-based peripheral nerve blocks (PNBs) specific for both above- and below-knee levels of amputation, and touches on the importance of continuous peripheral nerve blocks (CPNBs). This review will primarily concentrate on the effectiveness and impact of PNBs in cases of MLEA, and it does not intend to focus on the discussion of related studies concerning neuraxial anesthesia (NA). While the procedural aspect, including the ultrasound anatomy and step-by-step instructions, is essential for successfully performing PNBs, it is beyond the scope of this article; however, a brief overview of some important approaches for each potential PNB used is delivered to help bring their clinical value into context. The subsequent section illustrates the impact of RA on postoperative morbidity and mortality, perioperative analgesic management, and healthcare costs and resources. In addition, the most recent protocols and services employing RA in facilitating lower limb amputation will be examined. Finally, recent developments in surgical practices in the management of MLEA and post-amputation pain will be briefly summarized.

The objective of this review is to summarize current evidence regarding the efficacy of different RA techniques and their impact on perioperative outcomes and healthcare expenditures. It will also identify gaps in research concerning the superiority of RA in lowering postoperative mortality compared to alternative anesthesia modalities, as well as their particular influence over healthcare costs and resources. It will recommend a structured approach for perioperative pain management.

Methodology

This narrative review was initiated by gathering relevant English-written scholarly literature using data from the most recent publications (January 2018 to October 2024) in the form of original research articles, systematic reviews, and case reports from PubMed and Google Scholar online scientific databases using the keywords ‘lower extremity,’ ‘amputation,’ ‘regional anesthesia,’ ‘peripheral nerve blocks,’ ‘morbidity,’ ‘mortality,’ ‘phantom limb pain,’ ‘residual limb pain,’ ‘chronic post-surgical pain,’ ‘transitional pain service,’ and ‘enhanced recovery.’ Papers were evaluated based on their titles and abstracts, and then full-text articles were assessed. We then carefully evaluated references from key articles for additional sources. Although specific inclusion and exclusion criteria were not adopted, articles were prioritized according to their pertinence to our review theme and objective.

The analysis primarily included 24 articles with various study designs conducted in diverse geographical locations. These studies were categorized into the following themes: post-amputation pain, RA approaches, morbidity/mortality, and early recovery protocols or pain services involving RA. Additional articles from 2013, 2016, and 2017 on topics related to basic pain science were then added due to their foundational significance. Following that, we included articles from expert opinion series such as those offered by the American Society of Regional Anesthesia (ASRA) for in-depth discussion of relevant RA approaches. This article was adherent to the SANRA (Scale for the Assessment of Narrative Review Articles) checklist for improving reporting quality [7].

## Review

Manifestation of pain following limb amputation

Phantom Sensation and Phantom Limb Pain (PLP)

Up to 95% of amputation patients experience amputation-related pain, with 79.9% describing PLP and 67.7% reporting residual limb pain (RLP) regardless of the indication for amputation [8]. Phantom feelings are painless perceptions like itching or tingling that arise from a missing body part following deafferentation. In contrast, PLP, also known as post-amputation pain syndrome, manifests as a painful or unpleasant sensation in the location of the lost or defective body part [9].

After amputation, various pathophysiological changes occur at different levels: 1) Peripheral nerves: Trauma to neurons and tissues alters afferent and efferent impulses to and from the missing limb. Severed nerves may form neuromas and hyperexcite. 2) Spinal cord: Nerve hypersensitivity, neural activity, and neuronal receptive field increase with spinal cord central sensitization. The dorsal horn of the spinal cord has more N-methyl-D-aspartate (NMDA) activity, which makes it more open to substance P, tachykinins, and neurokinins. This makes the receptors more active. Neuronal change in the spinal cord can cause descending inhibitory fibers to lose their targets. 3) Brain: As the cortex reorganizes, adjacent regions in the primary somatosensory and motor cortex replace the severed area, causing pain and sensation in the missing limb [8]. According to a growing body of research, nerve changes, such as neuroma formation (a non-cancerous nerve tissue growth), contribute to stump and phantom pain after amputation. A recent cross-sectional study argued that swollen neuromas detected on ultrasound do not cause post-amputation pain. A positive Tinel sign, a simple diagnostic assessment that identifies irritated nerves by tapping to cause a tingling or 'pins and needles' sensation, occurs significantly more often in amputation patients who have pain, regardless of neuroma swelling. This backs up the traditional explanation that transected nerve endings play a major role in post-amputation pain [10].

Addressing the patient's pain behaviors may enhance the success of therapy [8]. PLP usually occurs in the bottom portion of the anatomically missing limb. The pain presentation may vary, although it is commonly characterized as cramping, burning, or shooting. A frequent observation is that if a patient has experienced intense pain in a limb prior to surgery, they will inevitably endure the same pain after the extremity is removed. Phantom pains often manifest as recurrent episodes lasting from just a couple of seconds to much longer periods of several hours. Fortunately, severe and unrelenting phantom pain is experienced by a minority of patients [3]. Approximately 75% of victims of PLP report its presence within the first week. Many patients notice phantom pain improvement over the first year, while in some cases, it completely resolves. However, many patients will end up with lifelong phantom pain. The risk factors for developing PLP include intense preoperative pain, bilateral amputation, stump pain, the history of several extremity operations, and advanced age. The occurrence of phantom pain does not exhibit any distinct disparity between genders, and psychological variables such as depression or anxiety are not determinants. Importantly, patients with significant psychological risk factors tend to encounter more intense phantom pain, accompanied by higher degrees of disability and dependence on pain medicine [3].

Residual Limb Pain

RLP or surgical stump pain is acute, sharp, and intense, and since there is considerable tissue trauma, it is mostly nociceptive pain. However, direct neurological injury also brings about a major neuropathic pain component [3]. Therefore, allodynia, defined as pain elicited by a stimulus that typically does not provoke pain, and hyperalgesia, characterized by an augmented pain response to a stimulus that ordinarily induces discomfort, should be investigated. Acute stump pain is anticipated to subside within the initial weeks following amputation; however, 10% of patients may develop chronicity. If someone has long-lasting stump pain, a number of different causes should be ruled out, such as infection, wound dehiscence, reduced blood flow, osteomyelitis, osteophytes, hematomas, inadequate muscle tissue coverage, and prostheses that don't fit properly [3]. RLP is often mild and characterized by pressing, throbbing, burning, squeezing, and stabbing sensations [8].

Chronic Post-amputation Pain (CPAP)

All three post-amputation pain phenomena may coincide and progress to chronicity in the same patient. Since a pain type is rarely completely isolated, a thorough evaluation of every patient is required to determine the primary pain type at that particular time in order to optimize treatment and reduce the risk of chronicity [3]. Studies have indicated that chronic pain is multifactorial and has a significant psychological component. Depression, anxiety, and elevated stress trigger both the intensity and chronicity of post-amputation pain [8]. Other circumstances that may impede post-amputation healing, recovery, or rehabilitation could predispose to chronicity. It was found that genetic variation may be pertinent to the development of CPAP [11].

It is advised to identify at-risk patients, specifically those who are younger, female, experiencing pre-operative chronic ischemic pain in the region of surgery, suffering from pre-operative chronic pain in other regions, and exhibiting symptoms of depression and anxiety, as well as those utilizing high doses of opioids pre-operatively [11]. There are four commonly used diagnostic criteria for CPAP: 1) Pain develops after surgery, or if pain existed before surgery, it persists or intensifies for three to six months after amputation. 2) Pain may occur in the residual limb, phantom limb, or surgery site. 3) Pain significantly impairs daily life, emotional health, or quality of life. 4) Other medical conditions or psychological factors cannot explain the pain [12].

Patient population and general considerations

The main causes of amputation include peripheral vascular conditions and diabetes, trauma, cancer, malignancy, and congenital disorders [8]. Data indicates that 93.4 % of all lower-extremity amputations result from vascular disease. Trauma accounts for 5.8 % of lower-limb amputations, yet it is the predominant cause during the second and third decades of life. Cancer constitutes 0.8 % of all amputations and is the leading cause among individuals aged 10 to 20 years [13]. Indications for lower extremity amputation are preserved for patients who suffer comorbidities, failed revascularization trials, sustain anatomical traits that impede revascularization, trigger tissue loss, or promote infection [14]. Statistics showed that the postoperative mortality rate for above-knee amputation (AKA) was 30 %, and for below-knee amputation (BKA), it was 14 % [15]. Postoperative mortality correlates with advanced age, dependent functional status, proximal amputation, history of cardiac disease, chronic renal impairment, preoperative infection, and corticosteroid usage [16-19].

Some common complications after amputation surgery include cardiopulmonary complications, prolonged ICU admissions, wound infections, and the need for revision surgery [20,21]. Severe comorbidities like renal, liver, or cardiovascular diseases, along with diabetes mellitus, are to blame for this [22]. According to estimates, 80% of patients with peripheral artery disease who require lower limb amputation have concurrent coronary artery disease, with certain individuals undergoing preoperative dual antiplatelet therapy, significantly raising their perioperative risk profile [23,24].

Advanced age results in an exaggerated body response to spinal and general anesthesia. This means that during surgery, older patients with multiple comorbidities, decreased cognitive, cardiac, and pulmonary capacity due to physiological changes, and impaired organ function are more likely to have anesthesia-related complications like prolonged mechanical ventilation and hemodynamic instability. They are also more likely to develop delirium or cognitive dysfunction after surgery. These patients are also more likely to require longer hospital stays and have higher rates of morbidity and mortality [25]. In situations involving emergency or urgent lower limb amputation, neuraxial blocks are avoided due to coagulopathy, systemic infection, and hemodynamic instability [26]. Furthermore, adverse effects like hypotension and respiratory depression have been linked to epidural catheters [27,28]. For these patients, PNBs may be effective. This is attributable to reduced hemodynamic alterations and the absence of motor impairment. Furthermore, early ambulation and improved pain management promote enhanced recovery in patients undergoing lower limb procedures with PNBs [29,30].

RA techniques and approaches

Relevant Anatomy

**Figure 1:**
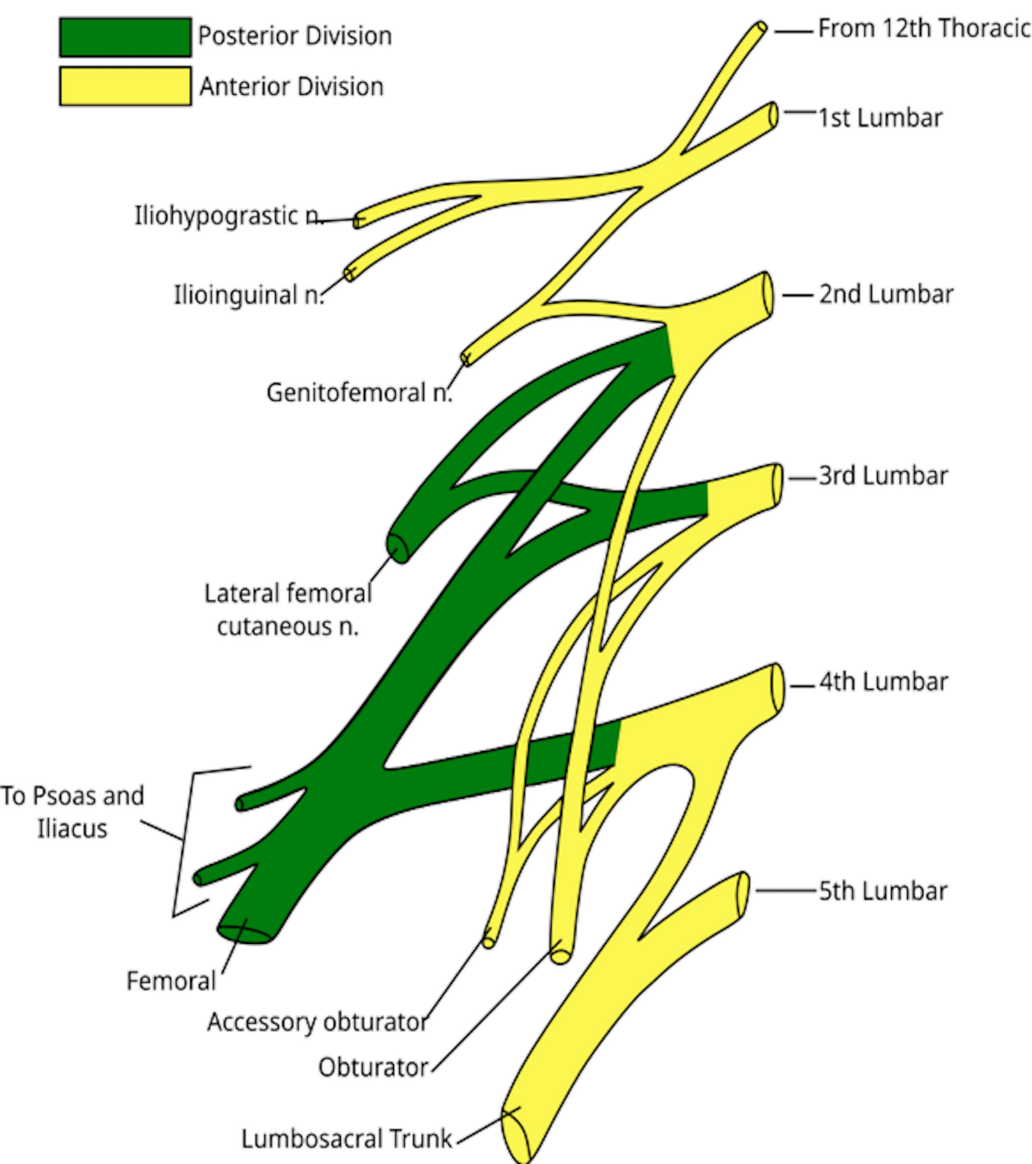
The lumbar plexus. Source: [33] (Licensed under CC BY 3.0; https://creativecommons.org/licenses/by/3.0/).

**Figure 2:**
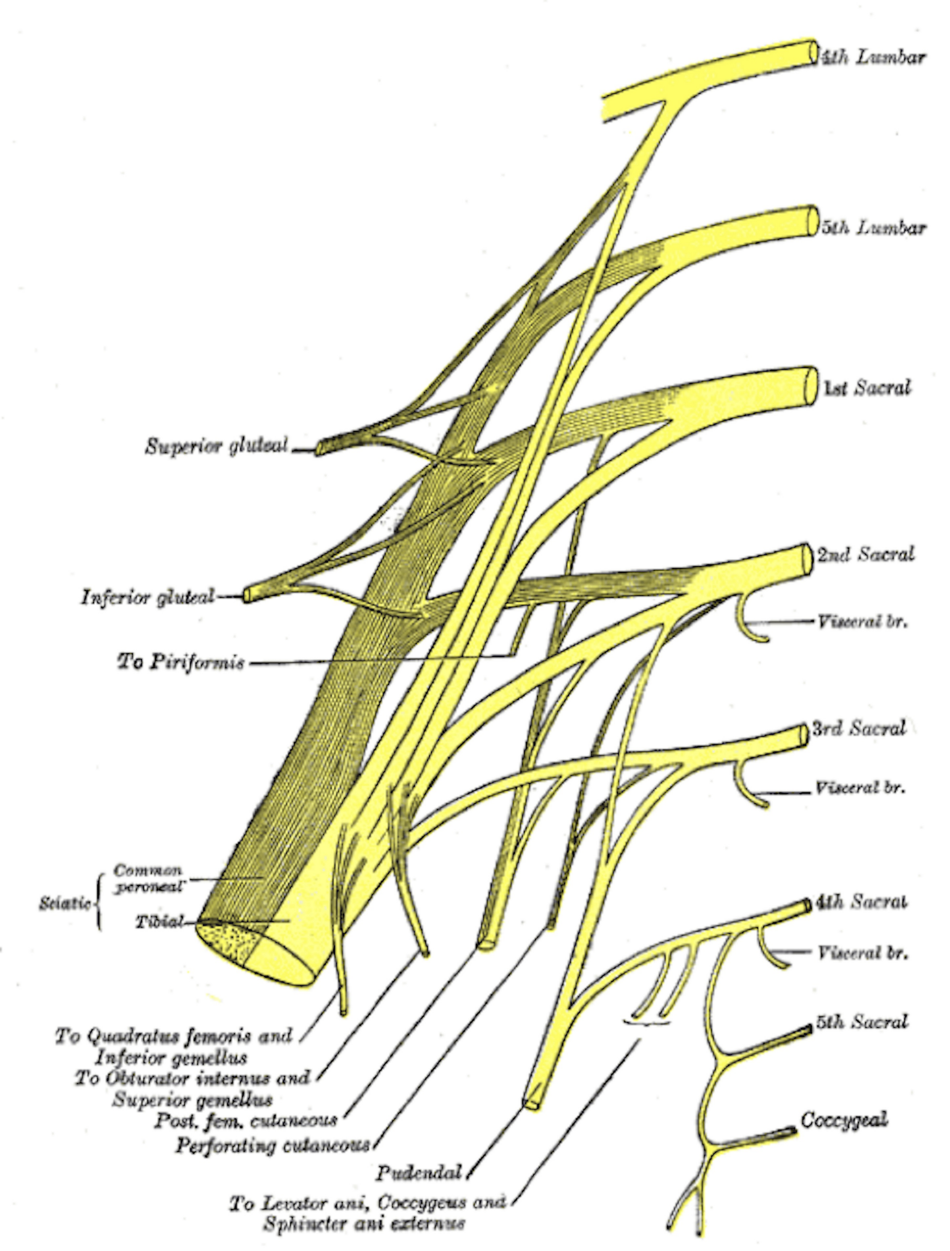
The sacral plexus. Source: [34] (Public domain)

**Figure 3:**
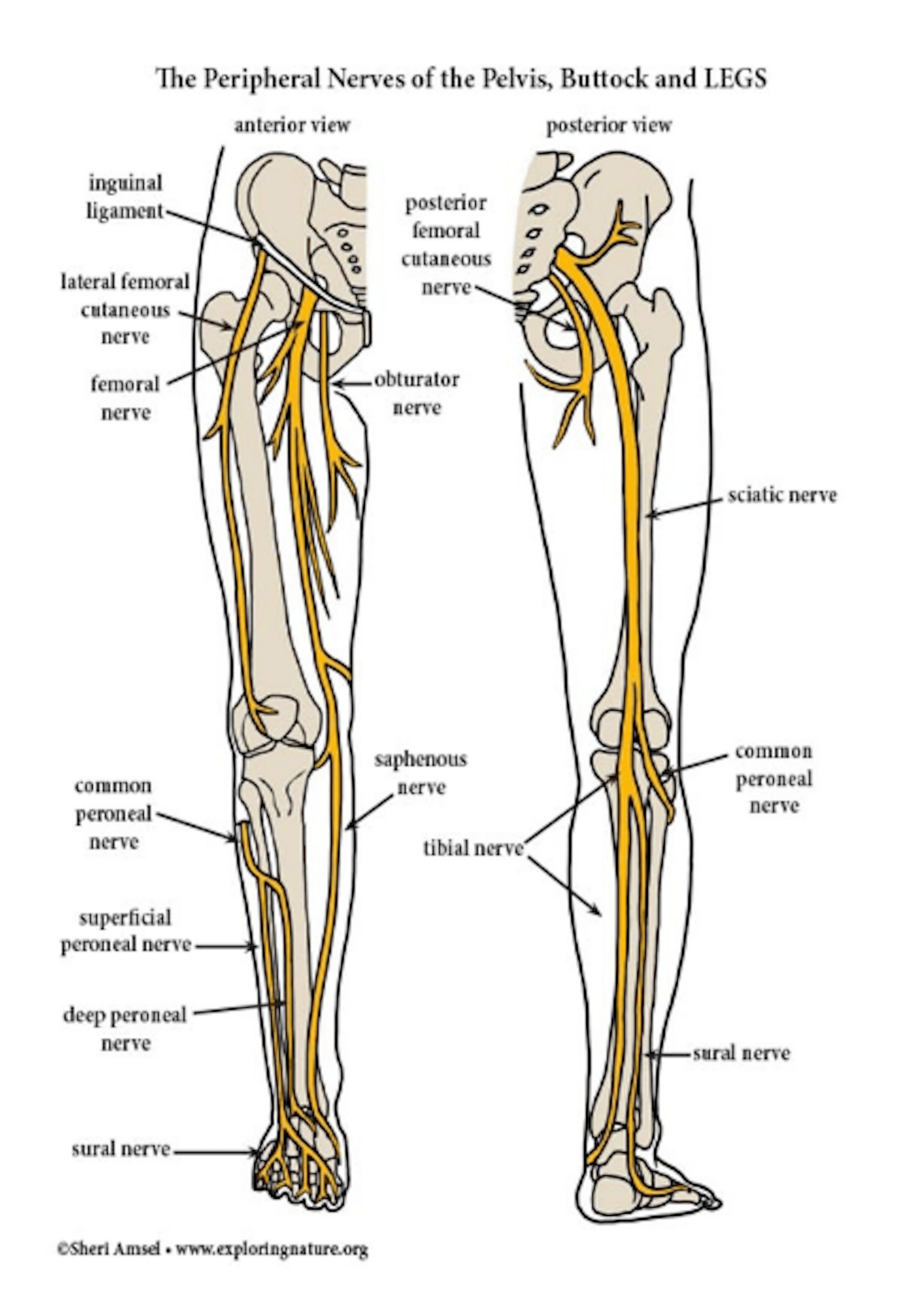
The peripheral nerve network of the right lower extremity: anterior and posterior views. Source: [35] Used with permission from Amsel, Sheri (Exploring Nature Educational Resource)

**Figure 4:**
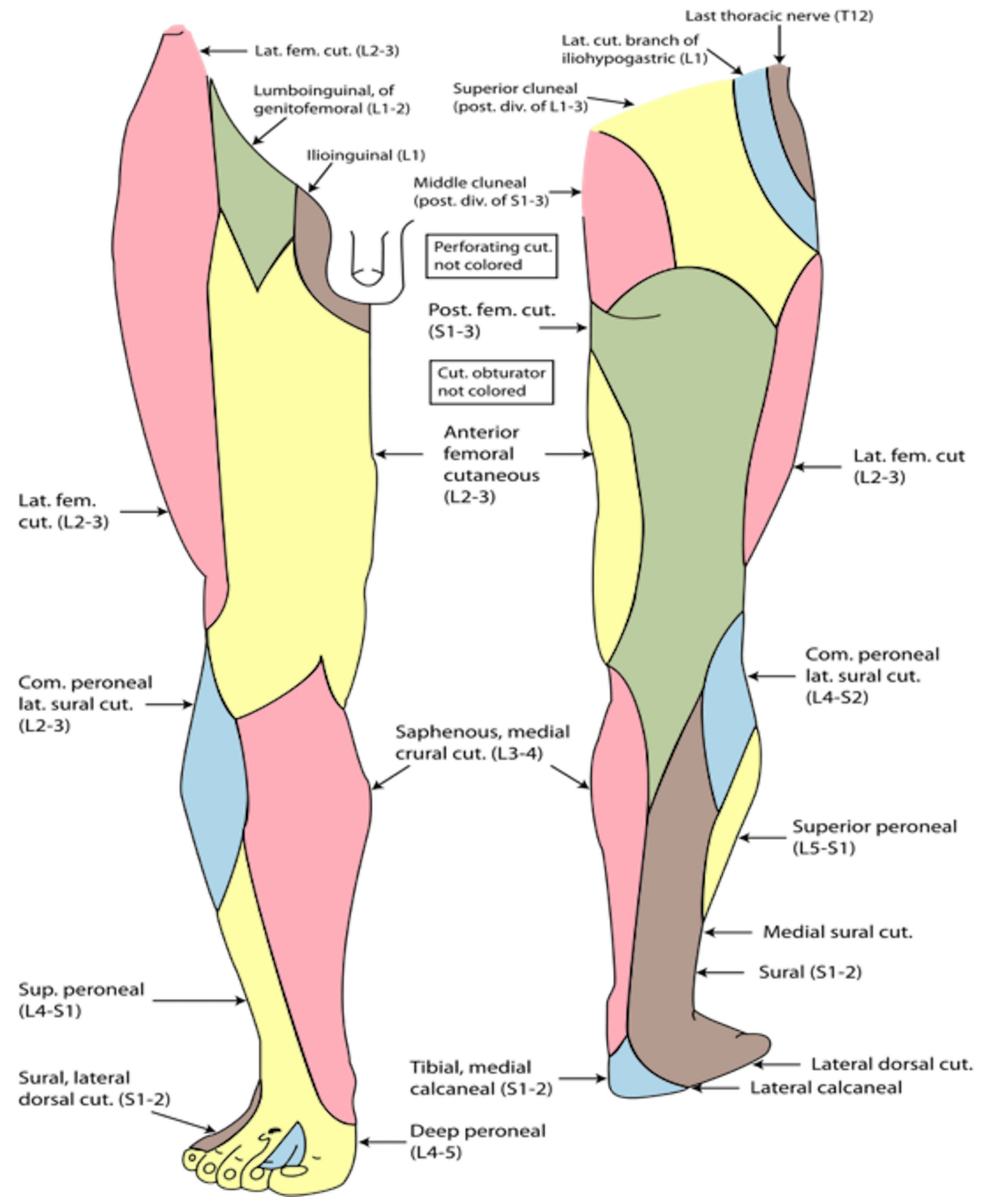
The cutaneous innervation of the right lower limb: anterior and posterior views. Source: [36] (Public domain).

The lumbar plexus arises from the ventral rami of the L1-L4 spinal nerves, along with a minor branch from the T12 nerve (Figure 1), whereas the sacral plexus (Figure 2) comprises the ventral rami of the L4, L5, S1, S2, and S3 nerve roots [31]. The lower extremity is innervated by the femoral nerve, originating from the posterior rami of the L2, L3, and L4 nerve roots; the largest branch of the lumbar plexus; the lateral femoral cutaneous nerve, arising from the posterior divisions of the L2 and L3 nerve roots; the obturator nerve, stemming from the anterior divisions of L2-L4; and the sciatic nerve, formed by major branches of the sacral plexus from L4 to S3 (Figure 3). The anterior thigh is sensory (Figure 4) and motor-innervated by the femoral nerve. The ipsilateral medial leg aspect receives sensory signals from the saphenous nerve, which is the terminal branch of the femoral nerve. Lateral thigh sensory innervation comes from the lateral femoral cutaneous nerve, which originates from the dorsal branches of L2-L3. The sensory and motor branches of the obturator nerve innervate the medial thigh and adductors, respectively. Obturator and lateral femoral cutaneous nerves do not provide sensory or motor innervation to the leg below the knee. The sciatic nerve gives motor innervation to the posterior aspect of the thigh, while the posterior cutaneous nerve of the thigh (S1-S3) provides sensory innervation to the posterior thigh aspect. The sciatic nerve bifurcates into the tibial and common peroneal nerves posteriorly, just above the popliteal fossa, providing sensory and motor innervation to the anterior, lateral, and posterior lower leg [31,32]. Thus, AKA is achieved by blocking the femoral, lateral cutaneous femoral, sciatic, and obturator nerves, while BKA is done by blocking the sciatic and femoral nerves.

Above-Knee Amputation

While blocking four nerves takes time, requires high local anesthetic doses, and increases the risk of local anesthetic systemic toxicity (LAST), patients with sepsis, thrombocytopenia, and severe respiratory infection underwent successful AKA procedures using high inguinal femoral, sciatic, and lateral cutaneous femoral blocks [37]. Obturator nerve blocks primarily serve as an analgesia and anesthesia component for comprehensive AKA. Notably, studies have reported effective primary nerve block anesthetics for those amputations that use only femoral and sciatic nerve blocks, but at the expense of increased sedation [38]. In AKA, the subgluteal approach is good to deeply block the sciatic nerve using a curvilinear probe, and the needle insertion point is located 4 cm caudally from the midpoint between the greater trochanter of the femur and the ischial tuberosity.

For AKA, a three-in-one femoral nerve block involves injecting a larger amount of anesthetic into the same area as a femoral nerve block, followed by applying caudal pressure to ensure the anesthesia reaches the femoral, obturator, and lateral cutaneous nerves in the block. Next, Labat's transgluteal approach for the sciatic nerve block is employed, which proximally blocks both the sciatic and posterior femoral cutaneous nerves. The landmark technique is used, which involves marking the cutting line between the greater trochanter and the posterior superior iliac spine 3 cm caudally and perpendicularly. However, partial block failures were observed when combining a three-in-one femoral block with a posterior approach for sciatic block [37,39,40].

The anterior sciatic block is ideal for continuous blockade due to its durable muscle layer, which ensures effective catheter stabilization while allowing the patient to ambulate. Moreover, the catheter is not impeding a thigh tourniquet. Additionally, the patient's leg requires no repositioning. However, ultrasound-guided sciatic nerve blockade via an anterior approach must always be conducted alongside nerve stimulation, given its depth at this level [41].

A lumbar plexus block targets the femoral, obturator, lateral femoral cutaneous, and genitofemoral nerves. However, this deep block, located in a highly vascularized region, results in a substantial volume of local anesthetics being absorbed more rapidly into the bloodstream; this presents an elevated risk of LAST. Hematoma can also occur more frequently in this highly vascularized region. A history of coagulopathy, or the use of anticoagulants or antiplatelet agents, significantly increases the risk of hematoma [38].

A recent case report demonstrated that using a lumbar erector spinae plane (ESP) block at the level of L3 for postoperative analgesia after an AKA causes the local anesthetic to spread to the roots of the lumbar plexus and results in good analgesia. However, additional research is required to ascertain the clinical relevance of lumbar ESP catheters in alleviating post-amputation pain [42].

Below-Knee Amputation

A combined adductor canal block, which targets the saphenous nerve, and popliteal sciatic nerve block, can ease pain during and after a BKA, and inserting CPNB catheters particularly enhances this effect [43]. A small prospective study examined high-risk patients who underwent BKAs without the use of a tourniquet. They found that an adductor canal saphenous nerve block and a popliteal sciatic nerve block, administered with a minimal dose of midazolam, were effective without requiring additional analgesics. This resulted in stable perioperative hemodynamics and overall patient satisfaction [44]. However, the tourniquet's ischemic pain and discomfort prevent it from serving as a sole anesthetic. Therefore, the use of femoral and popliteal sciatic nerve blocks is more comprehensive [45].

CPNBs and Their Significance

Concerning the pathophysiology of chronic PLP, it is proposed that cortical abnormalities may persist due to abnormal peripheral input. Performing ambulatory CPNBs for extended periods in patients with established PLP after extremity amputations may allow the cerebral cortex to organize more effectively by blocking peripheral pain signals for a longer period, therefore providing prolonged relief from PLP [46]. 

The optimal approach for managing post-amputation acute stump pain is RA, either by performing epidural infusions or PNBs [3]. Keeping this in mind, a newly conducted clinical trial concluded that a six-day CPNB alleviates PLP and mitigates physical and emotional dysfunction for a minimum of one month [46]. Numerous studies examining the application of RA for the prevention of PLP terminate neural blockade catheters within 48 hours post-surgery. Given that stump pain and the inflammatory process are still at their peak, it is evident that it is too early to terminate therapy. For that reason, it was found that perineural blockade should be maintained for at least 80 hours post-amputation to help the patient surpass the critical initial days of peak pain, thereby reducing sensitization. Some suggest that this is arguably the most vital intervention for acute pain management following amputation and is indispensable in reducing the probability of developing substantial PLP [3].

Effectiveness and outcomes

Impact on Postoperative Morbidity and Mortality

RA for lower limb surgeries in general is highly effective due to enhanced postoperative pain management, reduced opioid usage, less opioid-related side effects, and lower risks of respiratory-related problems [47]. As opposed to NA, patients on antiplatelet therapy may safely undergo BKA with dual femoral and sciatic nerve blocks [48]. Other case reports indicate that the combination of femoral and sciatic nerve blocks with minimal doses of fentanyl and/or midazolam is more effective than GA for stabilizing hemodynamics and alleviating postoperative pain in high-risk patients undergoing either AKA or BKA [49]. Another propensity-score matched observational study demonstrated that intraoperative hypotension, vasopressor administration, and postoperative ICU admission rates were reduced with RA compared to alternative anesthesia modalities; however, this study recruited patients who received spinal anesthesia or PNB in the RA group [50]. One later observational study that employed a more thorough propensity score analysis to control confounding factors was able to isolate the specific findings of PNBs and revealed that, in comparison to GA, PNBs are associated with reduced risks of pneumonia, acute kidney injury, and overall major complications after lower extremity amputation in diabetic patients with coagulopathy [51]. A different retrospective study demonstrated that combined femoral and sciatic nerve blocks administrated immediately prior to BKA significantly reduced the incidence of post-operative delirium compared to patient who underwent GA [52].

A large-scale study that looked back at cases of lower limb amputation found that patients who received RA had fewer pulmonary complications at 48 hours and within 30 days and fewer other major perioperative complications, like septic shock, blood transfusions, and reoperations compared to patients who underwent GA [53]. However, it did not find a significant difference in mortality rates between the two patient groups, consistent with findings from other studies [15,50]. The presence of multiple preexisting comorbidities among amputation patients may help to explain this finding [15].

On the other hand, the results of a single center study show that performing extremity amputations under PNBs, was associated with the least mortality/morbidity compared with GA and NA. It also showed a decreased need for postoperative intensive care, the mean length of hospital stay, and hospital costs compared with the other groups [54].

Several studies have demonstrated the effectiveness of CPNBs in reducing postoperative opioid consumption, although their evidence in contributing to the long-term relief of PLP and CPAP is limited [11]. Importantly, a recent retrospective cohort study employing extensive population-based databases revealed that the incidence rates of PLP post-amputation were, in descending order of frequency, GA, NA, and RA [9].

Impact on the Preemptive Multimodal Perioperative Analgesic Plan

Identifying high-risk patients and early involvement of acute pain service to optimize the analgesic regimen before lower limb amputation is crucial to prevent harmful central nervous system (CNS) sensitization. It was found that starting an analgesic regimen 48 hours before surgery reduced PLP at six months [55]. A recent systematic review of 37 randomized controlled trials indicates that RA, not confined to a specific block or surgical procedure, could decrease chronic post-surgical pain for up to six months following elective non-cardiac surgery, significantly reduce acute postoperative opioid consumption, and potentially lower the development persistent opioid use [56]. Thus, an assertive multimodal analgesic protocol incorporating early RA and ongoing chronic opioid therapy should be undertaken. Suddenly discontinuing the patient's baseline opioid requirement may lead to insufficient pain management and heighten the risk of acute withdrawal symptoms.

Multiple non-opioid adjuncts must be employed, as pain is modulated through multiple receptors. The multimodal strategy for preemptive analgesia in lower limb amputation should incorporate gabapentin, acetaminophen, nonsteroidal anti-inflammatory drugs (NSAIDs), and a suitable RA technique [43]. RA is the gold standard for managing acute RLP [3]. In the absence of RA, the intensity of RLP necessitates management with potent opioids as a baseline treatment. When administered alone, opioids often prove insufficient and require consumption in quantities that result in significant side effects. Intraoperative low-dose ketamine infusions, an NMDA receptor antagonist, may enhance postoperative pain scores, and intraoperative dexmedetomidine infusions may serve as a method to suppress nociceptive neurotransmission and deliver effective analgesia for patients exhibiting opioid tolerance, patients who did not receive RA or whose PNB is not working optimally, or those suffering from pain syndromes [57,58].

A retrospective study demonstrated that following combined femoral and sciatic nerve blocks, patients reported significantly reduced subjective pain scores at 6, 12, and 24 hours post-BKA compared to those who underwent GA for the same procedure. Additionally, their morphine milligram equivalent (MME) was markedly lower, and the incidence of postoperative nausea and vomiting was significantly reduced [52].

Research has been conducted on non-pharmacological interventions for managing post-amputation pain and RLP, presenting varying levels of evidence. These interventions include transcutaneous electrical nerve stimulation, mirror therapy, biofeedback, acupuncture, spinal cord stimulation, neuromodulation techniques, virtual and augmented reality, and sympathetic block, which may be utilized individually or in conjunction [8]. Nonetheless, a comprehensive examination of this subject exceeds the parameters of this paper.

Impact on Healthcare Costs and Resource Management

In 2019, the projected expense of a major amputation in the United States exceeded $89,000 per patient. With over 1.6 million individuals living with amputations, this burden is significant. Furthermore, over 50% of these amputations are major (transtibial or transfemoral), both of which possess significant potential for detrimental effects on mobility and autonomous life [2,14,59,60].

RA approaches, including NA and PNBs for post-amputation pain, can substantially influence healthcare costs and resource allocation. Cost reduction could follow from the effective management of pain; these techniques can decrease the necessity for opioid medications, leading to cost-effectiveness through reduced pharmaceutical expenses and a lower risk of opioid-related complications [52]. Research demonstrates that the incorporation of RA techniques into a preemptive multimodal analgesia strategy leads to reduced hospital stays [54]. The decreased occurrence of postoperative adverse events [53] and acute post-amputation pain [11], along with related complications, can result in decreased long-term healthcare expenditures. Moreover, improved pain management can elevate patient satisfaction and outcomes [44].

A good example of resource allocation achieved by the specific use of PNBs in MLEA is illustrated in a case report during the early stages of the coronavirus disease 2019 (COVID-19) pandemic that highlights the use of a four-block regional anesthetic technique (subgluteal sciatic, femoral, obturator, and lateral femoral cutaneous) as the primary anesthetic for an urgent AKA in a patient with severe respiratory symptoms, thereby preserving the need for mechanical ventilation in this high-risk group [61]. Despite the early costs associated with the application of RA approaches, the long-term benefits in cost reductions and resource efficiency can be substantial [62].

Modern practices implementing regional anesthesia to facilitate lower extremity amputation

Limb amputation is one of the procedures most likely to result in chronic post-surgical pain, alongside inguinal hernia repair, spine surgery, thoracotomy, and mastectomy [63,64]. The transitional pain service, emphasizing the long-term interdisciplinary management of surgical candidates predisposed to chronic pain and aiming to integrate postoperative, in-hospital, and outpatient care, has advocated for the extensive application of RA techniques due to their significant opioid-sparing effects [65-67]. A pilot study looked at the multidisciplinary perioperative Lower Extremity Amputation Protocol (LEAP) model for the enhanced recovery of vascular amputation patients, which included the standardized use of RA. RA was performed on 100 % of patients in the study group, whereas only 34 % of the control group received it. It was demonstrated that following this pathway, which is heavily reliant on RA, can significantly decrease postoperative length of stay and accelerate the timeline to independent ambulation with a prosthesis [68].

Advancements in surgical techniques

Recent developments in surgical practices are emerging in the multidisciplinary preventive and therapeutic management of MLEA and post-amputation pain, such as targeted muscle reinnervation, which was initially developed to enhance upper limb myoelectric prosthesis control, involving redirecting severed nerves to reinnervate specific muscles, thereby minimizing pain and neuroma formation. A further technique under assessment for MLEA cases is the regenerative peripheral nerve interface, which involves the implantation of free muscle grafts to function as biological interfaces for severed nerves, with the objectives of promoting nerve regeneration and preventing neuromas, thereby enhancing prosthetic integration and alleviating post-amputation pain [69].

Evaluation and boundaries

This overview critically analyzed five main recent papers [50,51,53,54,70] that focused on investigating the impact of RA on perioperative mortality rates in MLEA compared to other anesthetic modalities and reflected the extensive research landscape (Table 1). Their importance is highlighted for the following reasons: 1) Two of these articles were large-scale retrospective studies [53,70]. A notable strength of these two studies was their population-based setting, spanning a broad and diverse geographic area to mitigate selection bias; 2) Three retrospective cohort studies employed propensity score analysis to enhance data reliability by removing possible confounding factors such as patient demographics, comorbidities, medications, and surgical type [50,51,70]; 3) To consider the opposing perspective, only one retrospective analysis, including 203 patients at a single center in Turkey, showed that RA may decrease mortality rates after MLEA in comparison to GA [54]. However, it recruited a small number of patients, compared multiple anesthesia techniques, and included amputation cases for both the upper and lower extremities. It is noteworthy that all articles acknowledged RA for mitigating significant perioperative adverse events.

**Table 1:** Summary of studies that focused on investigating the impact of regional anesthesia on perioperative morbidity and mortality rates in major lower extremity amputations compared to other anesthetic modalities. LEA: lower extremity amputation, ACS NSQIP: American College of Surgeons National Surgical Quality Improvement Program, GA: General anesthesia, RA: regional anesthesia, NA: neuraxial anesthesia, PNB: peripheral nerve block, SA: spinal anesthesia, AKA: above-knee amputation, BKA: below-knee amputation, ICU: intensive care unit, EA: epidural anesthesia.

Study	Study Design	Population, Location and Study Period	Number of Patients and Study Groups n (n%)	Key Findings
Kurt (2022) [54]	Retrospective cohort study	UEA and LEA from a single center in Turkey between 2017 and 2021	Total patients = 203 (100%), PNB = 66 (32.5%), SA = 64 (31.5%), GA = 63 (31%), EA = 4 (2%), CSEA = 3 (1.5%), Sedo-analgesia = 3 (1.5%). UEA = 75 (37%), LEA = 128 (63%) PNB for UEA = 54 (71.5%) SA for LEA = 62 (48.9%)	Hospital stay (Mean ± SD, days): RA = 8.7 ± 7.4, GA = 15.0 ± 20.6 Mortality Rate: First 24 hours = 1 (0.5%), Next 48 hours = 1 (0.5%), Total 90-day mortality = 10 (4.9%), of which 6 (60%) occurred in the GA group. RA, especially PNBs reduce morbidity/mortality, postoperative intensive care, hospital stay, and hospital costs.
Kim et al. (2021) [51]	Retrospective cohort study using propensity score analysis	Diabetic patients with coagulation abnormalities who underwent LEA for diabetic foot ulcer from a single center in South Korea between January 2010 and December 2020	Total patients = 320 (100%), GA = 205 (64%), PNB = 115 (36%), Of which MLEA (BKA) after adjustment: GA = 27.2 (11.7%), PNB 16.7 (10.6%)	The adjusted analysis showed that PNB had lower pneumonia, acute kidney injury, and total major complications than GA. GA was also associated with more intraoperative crystalloid administration and vasopressor use.
Mufarrih et al. (2021) [53]	Retrospective cohort study	Patients undergoing LEA between 2005 and 2018, using data from the ACS NSQIP database	Total patients = 45492 (100%), GA = 40 026 (88.0%), RA (NA or PNB) = 466 (12.0%)	Pulmonary complications (48 hours): GA = 841 (2.1%), RA = 76 (1.4%) Pulmonary complications (30 days): GA = 2,521 (6.3%), RA = 323 (5.9%) Blood transfusions: Higher in GA than RA. Septic shock: Higher in GA than RA. Reoperation: Higher in GA than RA. Mortality rate: GA = 2281 (5.7%), RA = 389 (7.1%)
Kim et al. (2019) [50]	Retrospective cohort study using propensity score matching	Patients undergoing LEA with diabetes and/or peripheral vascular disease from medical records of a single center in South Korea between September 2007 and August 2017	Total patients = 519 (100%), GA = 227 (43.7%) SA = 239 (46.1%), PNB = 53 (10.2%), AKA (%): GA = 1.5%, RA = 1.5%, BKA (%): GA = 16%, RA = 16%, Minor amputation (%): GA =82.5%, RA = 82.5%	Adjusted results: 30-day mortality (%): GA = 3.5%, RA = 2.9% 90-day mortality (%): GA = 6.4%, RA = 4.6% Postoperative morbidity: No significant difference. Postoperative ICU admission (%): GA = 14.6%, RA = 7% Intraoperative hypotension (%): GA = 61.4%, RA = 14.6% Vasopressor use (%): GA = 52%, GA = 14%
Malik et al. (2018) [70]	Retrospective cohort study using propensity score matching	Patients underwent BKAs between January 1, 2007 and December 31, 2014, using the ACS NSQIP data set from 400 different sites	Total patients = 12723 (100%) GA = 10246 (80.5%), RA = 1835 (19.5%) Within the RA group: SA = 69.6%, PNB = 25% EA = 5.4%	Perioperative blood transfusion: GA = 16.5%, RA = 11.8% Postoperative complications (composite measure): GA = 29.1%, RA = 25.7% Mortality: No significant difference

Regarding limitations, some investigations included a small cohort of patients [50,51,54]. A recurrent theme identified in the examined literature was the inherent limitations associated with retrospective investigations, including insufficient patient data, the lack of randomization, and the potential for bias. Certain trials failed to address contraindications to RA, including patient refusal and local site infection. A few national registries utilized for gathering retrospective data provide only a restricted sum of postoperative follow-up days and offer limited comorbidity data, hence limiting the control of confounding variables [70]. The absence of randomization was an issue, as in some cases, AKA patients were excluded due to the difficulty in performing only RA on such critical cases [51]. Additionally, one other study mentioned that there existed a potential for bias originating from the preference of some anesthesiologists for specific types of anesthesia [15]. It was observed that the majority of studies fail to differentiate and analyze the impact of PNBs and NAs separately, instead categorizing them collectively as RA. This may cloud the results and hide potential differences in outcomes. In this context, of the three articles employing propensity score analysis in their methodology [50,51,70], only one specifically utilized it to separately analyze the role of PNBs on postoperative complications in amputation cases instead of considering RA as a whole [51].

This article has certain limitations intrinsic to the characteristics of a narrative review. Firstly, the selective inclusion of findings may lead to bias. Secondly, in contrast to systematic reviews with meta-analysis, there is an absence of quantitative analysis that highlights calculable outcomes across research. Lastly, the lack of a systematic approach may lead to the exclusion of pertinent studies or the undue emphasis on certain findings.

## Conclusions

MLEA is associated with significant perioperative morbidity and mortality, including complex pain manifestations that are often challenging to manage. Despite ongoing debate regarding the superiority of RA over other anesthetic modalities in reducing mortality after MLEA, evidence indicates that it is associated with fewer perioperative complications when compared to patients who received GA. To improve the quality of evidence and the reliability of findings, future multicenter studies employing a randomized prospective design with an extended follow-up period that specifically focus on PNBs are necessary to compare their mortality rates linked to MLEA to other types of anesthesia. Beyond that, a thorough investigation into the implications of RA on the healthcare costs and resources associated with MLEA is needed.

## Appendices

List of abbreviations

AKA: Above-knee amputation, BKA: Below-knee amputation, CGRP: Calcitonin gene-related peptide, CNS: Central nervous system, CPAP: Chronic post-amputation pain, CPNB: Continuous peripheral nerve block, COVID-19: coronavirus disease 2019, ESP: Erector spinae plane block, GA: General anesthesia, ICU: Intensive care unit, LAST: Local anesthetic systemic toxicity, LEAP: Lower extremity amputation protocol, MLEA: Major lower extremity amputation, MME: Morphine milligram equivalent, NA: Neuraxial anesthesia, NMDA: N-methyl-D-aspartate, NSAIDs: Nonsteroidal anti-inflammatory drugs, PNB: Peripheral nerve block, PLP: Phantom limb pain, PONV: Postoperative nausea and vomiting, RA: Regional anesthesia, RLP: Residual limb pain, RPNI: Regenerative peripheral nerve interface, SCS: Spinal cord stimulation, TENS: Transcutaneous electrical nerve stimulation, TMR: Targeted muscle reinnervation, TPS: Transitional pain service.
